# Microbiological assessment of the indoor air quality of a university health centre in Nigeria

**DOI:** 10.1186/2047-2994-4-S1-P51

**Published:** 2015-06-16

**Authors:** OO Ayepola, LO Egwari, GI Olasehinde

**Affiliations:** 1 Biological Sciences, Covenant University, Ota, Nigeria

## Introduction

The air within the healthcare environment may serve as a reservoir for microorganisms thereby contributing to the rate of infection. Regular monitoring of the microbial burden is necessary to ascertain the microbiological quality of hospital environments.

## Objectives

This study was conducted to evaluate the quality of the indoor air in a university health centre.

## Methods

The air microflora was assessed using the settle plate method. Air samples were taken from the waiting room, consulting rooms, nurse station, male and female wards as well as the laboratory to detect bacterial and fungal flora. The antimicrobial activity of five commonly used disinfectants was tested on some of the isolated bacteria.

## Results

Thirteen bacterial genera and two fungal genera were identified. The predominant bacteria were *Klebsiella* spp (15.7%), *Bacillus* spp (15.7%) *and Streptococcus* spp (10.5%). Among the less common bacterial isolates were *Staphylococcus aureus* and *Clostridium* spp*.* The fungal isolates include *Aspergillus niger* (50%) and *Mucor* spp (50%). The microbial burden was highest in the wards, followed by the consulting rooms and the waiting room. The antimicrobial activity the disinfectants varied with the concentrations tested. *Klebsiella* species were resistant to two of these disinfectants at all concentrations.

## Conclusion

The findings of this study revealed the presence of possible pathogens. This emphasizes the importance of regular air surveillance and proper infection control practices in hospitals.

## Disclosure of interest

None declared.

